# Facile Synthesis of Dual-Functional Cross-Linked Membranes with Contact-Killing Antimicrobial Properties and Humidity-Response

**DOI:** 10.3390/molecules29102372

**Published:** 2024-05-17

**Authors:** Ioanna Tzoumani, Denisa Druvari, Miltiadis Evangelidis, Alexios Vlamis-Gardikas, Georgios Bokias, Joannis K. Kallitsis

**Affiliations:** 1 Department of Chemistry, University of Patras, GR-26504 Patras, Greece; druvari@upatras.gr (D.D.); up1073714@ac.upatras.gr (M.E.); avlamis@upatras.gr (A.V.-G.); bokias@upatras.gr (G.B.); 2 Foundation for Research and Technology-Hellas, Institute of Chemical Engineering Sciences (FORTH/ICE-HT), Stadiou Street, GR-26504 Patras, Greece

**Keywords:** antimicrobial coatings, quaternary ammonium compounds, 2-(dimethylamino)ethyl methacrylate, cross-linking reaction, humidity responsive membranes, non-contact switches, actuators

## Abstract

Poly(2-hydroxyethylmethacrylate-co-2-(dimethylamino)ethyl methacrylate), P(HEMA-co-DMAEMAx), copolymers were quaternized through the reaction of a part of (dimethylamino)ethyl moieties of DMAEMA units with 1-bromohexadecane. Antimicrobial coatings were further prepared through the cross-linking reaction between the remaining DMAEMA units of these copolymers and the epoxide ring of poly(*N*,*N*-dimethylacrylamide-co-glycidyl methacrylate), P(DMAm-co-GMAx), copolymers. The combination of P(HEMA-co-DMAEMAx)/P(DMAm-co-GMAx) copolymers not only enabled control over quaternization and cross-linking for coating stabilization but also allowed the optimization of the processing routes towards a more facile cost-effective methodology and the use of environmentally friendly solvents like ethanol. Careful consideration was given to achieve the right content of quaternized units, qDMAEMA, to ensure antimicrobial efficacy through an appropriate amphiphilic balance and sufficient free DMAEMA groups to react with GMA for coating stabilization. Optimal synthesis conditions were achieved by membranes consisting of cross-linked P(HEMA78-co-DMAEMA9-co-qDMAEMA13)/P(DMAm-co-GMA42) membranes. The obtained membranes were multifunctional as they were self-standing and antimicrobial, while they demonstrated a distinct fast response to changes in humidity levels, widening the opportunities for the construction of “smart” antimicrobial actuators, such as non-contact antimicrobial switches.

## 1. Introduction

Microbial infection represents a substantial global health issue, affecting the overall well-being of individuals on a global scale. The invasion and proliferation of microorganisms, such as bacteria, viruses, fungi, or parasites within the body, leads to various diseases and health challenges. The increasing prevalence of antibiotic-resistant bacterial strains is a significant concern for researchers. Consequently, there is a high interest in creating surfaces and coatings that can efficiently diminish the presence of viable viral pathogens. A step further is to design and deploy touchless switches based on responsive materials that can effectively contribute to the reduction of microorganisms, therefore preventing cross-infection [1,2]. These innovations are sought after for use in various applications with minimal human exposure to infectious pathogens [3,4]. Various polymers with antimicrobial properties against bacteria, yeast, and viruses are studied as potential candidates for the development of coatings that can effectively prevent bacterial growth when applied to surfaces like glass, paper, plastic, and metal [5]. Regarding their mechanism of action, antimicrobial polymeric coatings can be classified into two main categories: (i) contact-killing surfaces with immobilized (via covalent bonds) antimicrobials that eliminate microorganisms after direct contact and (ii) polymeric compounds containing releasable antimicrobials, such as metal nanoparticles, and low-molecular-weight biocides or antibiotics [6]. Although the second approach is usually more efficient, the first category is more challenging since it requires careful selection of the antimicrobial species and the maintenance of a suitable hydrophobicity/hydrophilicity balance to achieve longer-lasting activity. In contact-killing coatings, the most employed antimicrobial agents are quaternary ammonium compounds (QACs), antimicrobial peptides (AMPs), and antimicrobial enzymes (AMEs) [7,8]. Polymers containing quaternary ammonium groups have gained significant attention recently as macromolecular antimicrobial agents mimicking the mode of action of antimicrobial peptides. These polymers derive their biocidal properties from positively charged ammonium moieties, with factors like alkyl chain length, halogenation, and ammonium group content affecting their effectiveness. Amphiphilic polymers are particularly studied for their advantages over cationic polymers, showing effectiveness against both Gram-positive and Gram-negative bacteria [9]. Notably, poly(dimethylaminoethyl methacrylate) (PDMAEMA) is significant for its potential quaternization, leading to tunable properties in derived cationic polymers like quaternized dimethylaminoethyl methacrylate (qDMAEMA) [10]. Thus, the cationic polymers derived from quaternized dimethylaminoethyl methacrylate (qDMAEMA) have garnered significant attention within the scientific community due to their tunable properties. The general structure of qDMAEMA consists of a hydrophilic ammonium cation and a hydrophobic alkyl chain. The amphiphilic nature of these polymers is crucial for their bactericidal effect, as their tertiary amino groups can be quaternized, resulting in positive charges that may interact with the negatively charged bacterial cell wall [11]. Different antimicrobial polymeric materials based on quaternized PDMAEMA functionalities have been employed [10,12,13] for the fabrication of coatings, including the growth of polymeric brushes from a surface [14,15] or mixing of quaternized PDMAEMA functionalities with a film-forming matrix [16]. As a general observation, the hydrophobic/hydrophilic balance is crucial for effective antimicrobial action since PDMAEMA chains quaternized with hydrophobic long alkyl groups are less active compared to less hydrophobic quaternized analogs.

In our group, we have developed a methodology for the preparation of cross-linked antimicrobial coatings and self-standing membranes based on the reaction of the epoxy ring of glycidyl methacrylate (GMA) with complementary units like acrylic acid [17,18]. These membranes exhibit antimicrobial properties through a combination of contact killing and releasing mechanisms of immobilized and electrostatically bound QACs biocides. Additionally, similar membranes have demonstrated humidity-responsive behavior, showing bending and flipping motions in response to moisture [19]. 

Remarkable progress has been made recently in the development of humidity actuators and sensors, employing a diverse range of materials such as natural polymers, synthetic polymers, and carbon-based materials [20,21,22]. To fabricate a humidity sensor/actuator, it is crucial to use materials that exhibit high affinity to water and hydrophobic properties at the same time. Achieving optimal sensing performance, characterized by high sensitivity, rapid response/recovery, and minimal hysteresis, necessitates a delicate equilibrium in material selection. The primary categories of humidity-responsive sensors/actuators include multilayer actuators [23], which leverage the differences in hydrophilicity between materials or layers to induce a response, and monolayer actuators [24], which incorporate one or more hydrophilic materials in their structure and react to asymmetrical stimuli. Furthermore, humidity-sensitive materials like hydrogels, cellulose nanofibers, etc., can be combined with other humidity-inert materials to form humidity-responsive bilayer actuators, enhancing control over the actuators’ deformations [25].

Building upon our previous findings, the present work expanded on the variety of polymeric structures and optimization of the processing routes applied in the membranes’ development towards a more facile, cost-effective, and environmentally friendly methodology, avoiding halogenated solvents. Our goal was to design stable biocidal surfaces based on contact-killing mechanisms. We utilized quaternized DMAEMA units to adjust the composition of amphiphilic random copolymers P(HEMA-co-DMAEMAx), optimizing hydrophobic/hydrophilic balance and antimicrobial activity by quaternizing DMAEMA units with long aliphatic chains. Incorporating 2-hydroxyethyl methacrylate (HEMA) not only controlled water solubility but also enhanced biofriendliness due to its biocompatibility and low cytotoxicity [26,27,28,29]. DMAEMA monomer was chosen in this study not only for its potential as an antimicrobial compound but also for its ability to react with other active monomers, such as the epoxy ring of GMA [30]. The outcome of this study provides insights into how the hydrophobic/hydrophilic balance of cationic polymers may affect antibacterial efficacy, offering insights for developing long-lasting antimicrobial materials. The resulting self-standing membranes with antimicrobial and humidity-responsive properties hold promise for applications in biomedicine, sensors, and contactless switches.

## 2. Results and Discussion

### 2.1. Synthesis and Characterization of Copolymers

To study the humidity responsiveness and antibacterial properties of cross-linked membranes, various copolymers of P(HEMA-co-DMAEMAx) were initially synthesized, and the optimal conditions for cross-linking with P(DMAm-co-GMAx) copolymers were determined. Subsequently, P(HEMA-co-DMAEMAx) copolymers were modified with varying contents of hexadecyl-ammonium groups, and their antibacterial activity was investigated along with their cross-linking reaction with complementary copolymer P(DMAm-co-GMA40). 

**Scheme 1:**
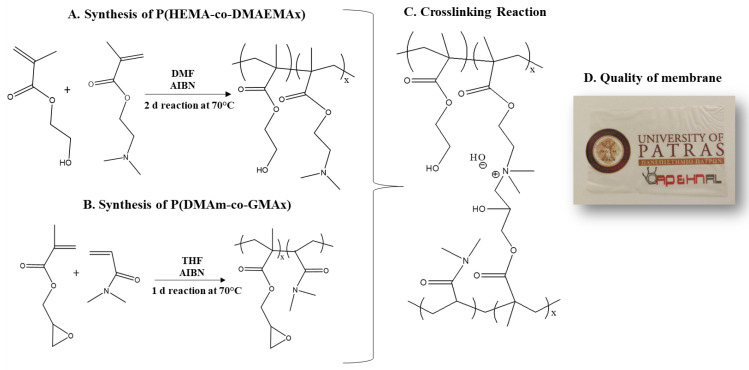
(**A**) Synthetic routes for the preparation of copolymers P(HEMA-co-DMAEMAx), (**B**) P(DMAm-co-GMAx), and (**C**) cross-linking reaction between them. The cross-linked membrane (**D**) is placed on top of a paper printed with logos to illustrate its transparency and homogeneity.

The synthetic routes followed for the synthesis of the copolymers and cross-linking reaction are depicted in Scheme 1.

**Figure 1:**
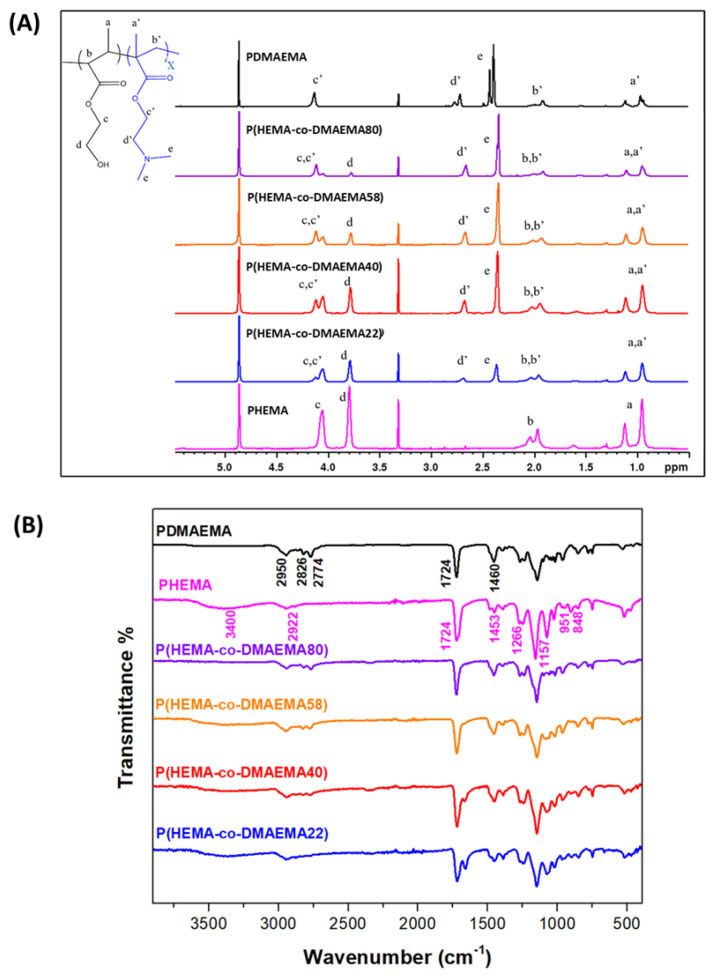
(**A**) ^1^H-NMR spectra in MeOD and (**B**) ATR-FTIR spectra of the P(HEMA-co-DMAEMAx) copolymers synthesized in this work, as well as of the respective homopolymers PHEMA and PDMAEMA for comparison.

First, P(HEMA-co-DMAEMAx) copolymers were synthesized through free radical polymerization with different % mol content of DMAEMA units, Scheme 1. To the best of our knowledge, the synthesis of P(HEMA-co-DMAEMAx) copolymers has been reported once in the literature using different polymerization conditions [31], where it was used for the development of a microfiltration membrane. The chemical composition of the synthesized copolymers was determined through ^1^H-NMR spectroscopy (Figure 1A) in MeOD. In the ^1^H-NMR spectra of the P(HEMA-co-DMAEMAx) copolymers, the signals at 0.80–1.20 ppm are attributed to the methyl protons (a, a′) of HEMA and DMAEMA units. The protons of the backbone (-CH_2_-, b, b′) of HEMA and DMAEMA can be identified at the area 1.80–2.20 ppm. The peak at 3.80 ppm is attributed to the methylene protons (d) of HEMA, while the peaks at 4.00–4.20 ppm correspond to the methylene protons of HEMA and DMAEMA (c, c′). The peak observed at 2.70 ppm is attributed to the methylene protons of PDMAEMA (d′). The presence of DMAEMA moieties in the final copolymers is also confirmed by the characteristic peak at 2.35 ppm, which corresponds to the methyl protons (e, e′) connected with the nitrogen atom in the DMAEMA units. Finally, the monomers’ ratio in the copolymers was calculated using the characteristic peaks (d) and (d′) of the two monomer units HEMA and DMAEMA, respectively.

Figure 1B shows the ATR-FTIR spectra of the P(HEMA-co-DMAEMAx) copolymers and the corresponding homopolymers PHEMA and PDMAEMA. The broad peak located at ~3400 cm^−1^ and the peak at 2946 cm^−1^ are attributed to the stretching vibration band of the hydroxyl group (-OH) and the aliphatic C-H stretching vibrations of HEMA, respectively. The bands at 1453, 951, and 848 cm^−1^ correspond to the deformation of methyl and methylene groups. The distinctive absorption bands of the PDMAEMA homopolymer can be attributed to the C-H(-N(CH_3_)_2_) stretching vibrations falling within the range of 2774–2950 cm^−1^ and the N(CH_3_)_2_ deformational stretching vibrations at approximately 1460 cm^−1^. The characteristic peak at 1724 cm^−1^, in both homopolymers and all the copolymers, corresponds to the stretching vibrations of the carbonyl C=O group, whereas the absorption bands which are observed at 1157 and 1266 cm^−1^ are characteristic of the C-O-C group. Furthermore, the band at 2826 cm^−1^ is attributed to the C-H stretching vibration of N(CH_3_)_2_ moieties, while the band at 1020 cm^−1^ corresponds to the C-N stretching vibration of a tertiary amine, confirming the presence of DMAEMA segments in the copolymers. As the concentration of DMAEMA decreases from P(HEMA-co-DMAEMA80) to P(HEMA-co-DMAEMA22), the C-N band’s intensity also reduces. 

Amphiphilic, random copolymers based on the hydrophobic glycidyl methacrylate (GMA) monomer and the hydrophilic, non-ionic *N*,*N*-dimethylacrylamide (DMAm) monomer were synthesized next. Polymerization reactions were carried out at 70 °C in tetrahydrofuran (THF), using AIBN as the initiator, following the previously reported procedure [32]. Schematically, the synthesis of P(DMAm-co-GMAx) copolymers (where x = 20, 40% mol GMA) is shown in Scheme 1. The P(DMAm-co-GMAx) copolymers were characterized by ^1^H-NMR and ATR-FTIR spectroscopies as described in the Supplementary Materials section, Figure S1A,B, and are in agreement with previous results [32].

### 2.2. Cross-Linking Reaction of Copolymers

The polymeric mixtures P(HEMA-co-DMAEMAx)/P(DMAm-co-GMAx) where x = 23 or 42% mol GMA were formed through the reaction between the amine groups of the DMAEMA unit and the epoxy groups of the GMA unit, a reaction reported recently by our group [30], facilitated by heat treatment at 120 °C for a duration of 24 h (Scheme 1).

Several pairs of complementary copolymers were explored (Table 1), and the composition of the copolymer mixtures was adjusted to cover various mixing ratios (r = nDMAEMA/nGMA, where nDMAEMA and nGMA are the equivalents of the two structural units, respectively). The range of mixing ratios included DMAEMA-rich mixtures (r = 6.6/1 or 3.5/1) as well as GMA-rich mixtures (r = 0.8/1 or 0.4/1). These mixtures were dissolved in ethanol, and the membranes were obtained through solvent casting at room temperature. The cross-linking reaction then took place in a solid state at various temperatures (room temperature, 80 °C, or 120 °C). The selection of complementary copolymers aimed at achieving DMAEMA-rich mixtures and stable cross-linked membranes with low swelling in water.

**Figure 2:**
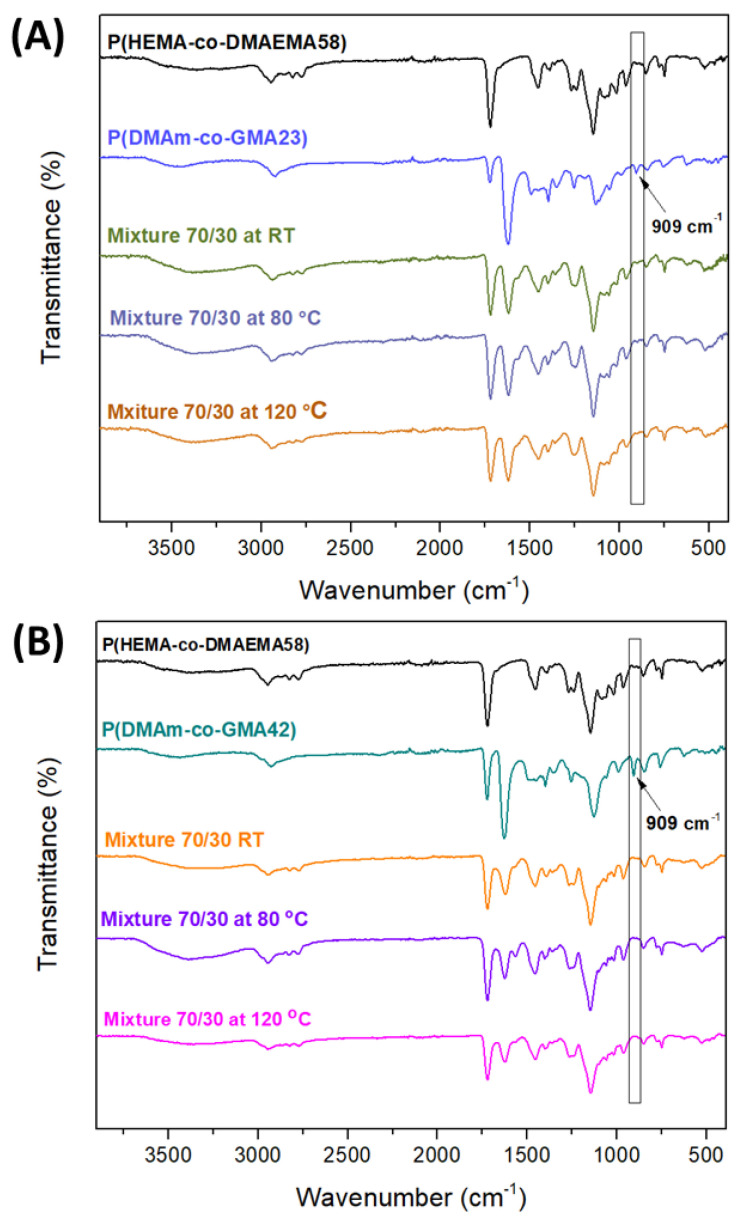
ATR-FTIR spectra of the (**A**) P(HEMA-co-DMAEMAx)/P(DMAm-co-GMA23) and (**B**) P(HEMA-co-DMAEMAx)/P(DMAm-co-GMA42) blend membranes after curing at RT, 80 °C, and 120 °C. The spectra of the respective copolymers are shown as well for comparison.

A first indication of the reaction progress was gathered from ATR-FTIR characterization of the membranes following thermal treatment. Attention was mainly given to the examination of the absorption peak associated with epoxy groups at 909 cm^−1^. Representative examples include the ATR-FTIR spectra of two membranes treated at room temperature, 80 °C, or 120 °C for 24 h, as depicted in Figure 2. Notably, the peak at 909 cm^−1^ either completely disappears (Figure 2B) or undergoes a significant reduction (Figure 2A), suggesting that the nucleophilic attack of the tertiary amine has led to practically quantitative epoxy ring opening. Thus, a result driven from the ATR-FTIR spectra is that the higher temperature favors the cross-linking reaction between the two copolymers.

Measurements of soluble fractions (%) of the polymeric membranes when immersed in water indicated that the membranes treated at 120 °C were more stable than the corresponding membranes treated at 80 °C or left at RT (room temperature) (Figure S2). This suggests that the cross-linking reaction between the complementary copolymers P(HEMA-co-DMAEMAx) and P(DMAm-co-GMAx) occurred to a satisfactory degree after thermal treatment at 120 °C since the soluble fraction remains low in most cases, from 0.5 to 16%.

Additionally, when comparing Figures S2B and S2D, it is revealed that membranes prepared with the copolymer P(DMAm-co-GMA23) exhibited a higher water uptake ratio (membranes M9-M12, approximately 105%) than those with P(DMAm-co-GMA42) (membranes M13-M16, approximately 85%), indicating that the swelling ratio of the cross-linked membranes can be affected by the GMA content in the copolymer. Thus, to attain more stable polymeric materials, membranes of blends P(HEMA-co-DMAΕΜAx)/P(DMAm-co-GMA42) 70/30 *w*/*w*% were selected for further studies, after cross-linking at 120 °C.

### 2.3. Quaternization of P(HEMA-co-DMAEMAx) Copolymers

**Scheme 2:**
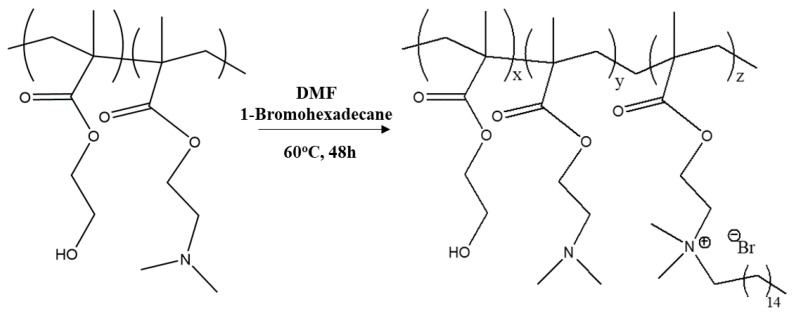
Quaternization reaction of the P(HEMA-co-DMAEMAx) copolymers with 1-bromohexadecane.

After the investigation of the development of cross-linked membranes P(HEMA-co-DMAEMAx)/P(DMAm-co-GMAx) with high stability, the next step towards creating antibacterial coatings was to introduce quaternary ammonium groups to the polymeric materials. In this context, a quaternization reaction of DMAEMA units took place, using 1-bromohexadecane in various compositions (Scheme 2). Aiming to acquire a deeper understanding of the hydrophilic/hydrophobic balance role on the antibacterial activity of the polymeric materials, three copolymers P(HEMA-co-DMAEMAx) with different DMAEMA content (x = 22, 58, 80) were selected to be quaternized. Thus, P(HEMAx-co-DMAEMAy-co-qDMAEMAz) terpolymers were finally obtained with different quaternization degrees of DMAEMA, as shown in Table 2.

**Figure 3:**
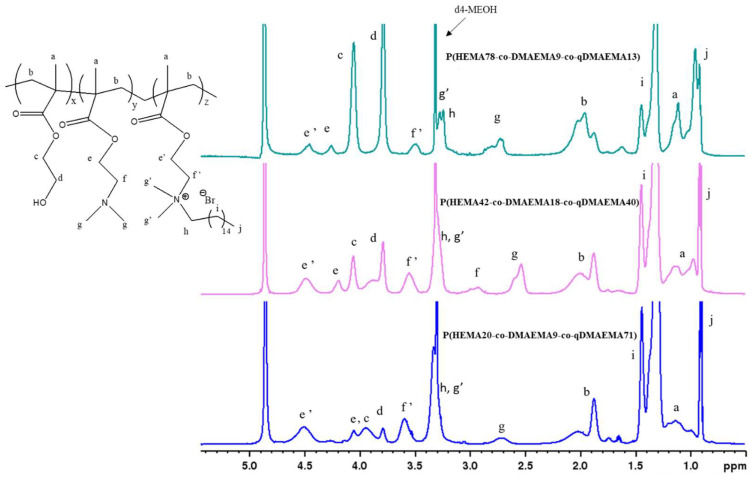
^1^H-NMR spectra of the quaternized terpolymers P(HEMAx-co-DMAEMAy-co-qDMAEMAz) in MeOD.

The terpolymers were characterized by ^1^H-NMR spectroscopy using MeOD to determine the degree of quaternization. Specifically, as shown in Figure 3, the successful reaction was evidenced through the appearance of the characteristic methyl peaks of the hexadecyl chain (-CH_2_-) at 1.2–1.5 ppm (i), compared to the spectra that appeared in Figure 1A. Moreover, some differences are observed in terms of the chemical shifts of some signals. Namely, the signal at 4.5 ppm corresponding to methylene protons of PDMAEMA (e′) has undergone a shift compared to the respective methylene peak (e) at 4.0–4.2 ppm, as a result of the quaternization of the pendant amino group. Additionally, protons corresponding to signals g′ (3.3 ppm) and f′ (3.6 ppm), which are linked to the nitrogen atom, exhibited a shift to the region of higher frequencies than the respective protons of the non-quaternized precursor. Moreover, the degree of quaternization was calculated using the peaks e′ and g of the qDMAEMA and DMAEMA units, respectively.

**Figure 4:**
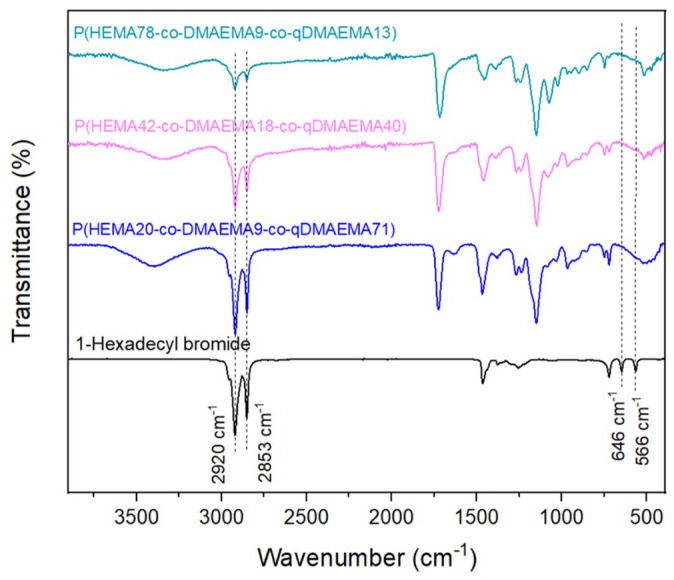
ATR-FTIR spectra of the quaternized terpolymers P(HEMAx-co-DMAEMAy-co-qDMAEMAz) and the 1-bromohexadecane as well, for comparison.

ATR-FTIR spectroscopy (Figure 4) indicated the successful introduction of the hexadecyl chain into DMAEMA units by the appearance of the peaks at 2920 and 2853 cm^−1^ in the P(HEMAx-co-DMAEMAy-co-qDMAEMAz) terpolymers’ spectra, which are attributed to the -CH_2_- groups of the long aliphatic chain. Notably, as the degree of quaternization increases, the intensity of the two peaks increases as well. Additionally, the disappearance of 646 and 566 cm^−1^ peaks, which are attributed to the C–Br bond of 1-bromodecahexane, is another indication of the quaternization reaction between the alkyl halide and the tertiary amine groups.

**Figure 5:**
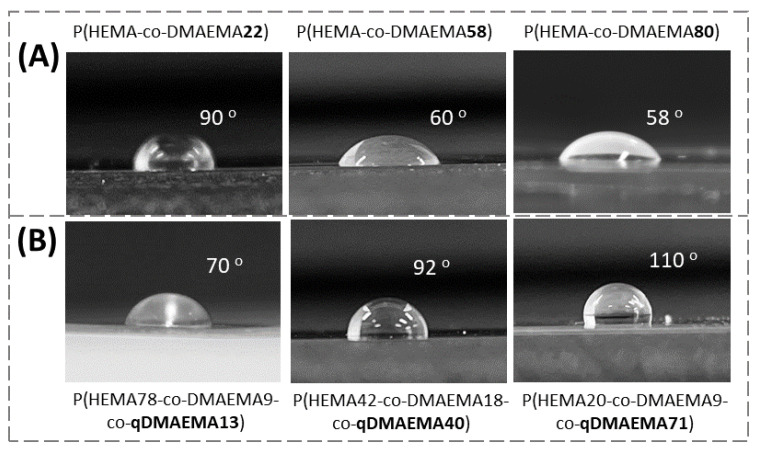
(**A**) Contact angle measurements of the individual starting copolymers P(HEMA-co-DMAEMAx) and (**B**) their quaternized analogs P(HEMAx-co-DMAEMAy-co-qDMAEMAz) in the form of coatings.

As described earlier, the main idea of this work was to develop cationic polymeric materials with antibacterial efficiency and investigate how the hydrophobicity/hydrophilicity balance affects their effectiveness. To study the surface wettability of the synthesized copolymers and their quaternized analogs, measurements of water contact angle on the respective polymers in the form of coatings were performed. As can be observed in Figure 5A, increasing DMAEMA content in the P(HEMA-co-DMAEMAx) copolymers enhanced the hydrophilicity of the copolymers’ surface. This was explained in view of the decrease in the contact angle values (from 90° to 58°). However, after the quaternization reaction, a different behavior was observed regarding the content of the quaternized DMAEMA unit in the P(HEMAx-co-DMAEMAy-qDMAEMAz) terpolymers (Figure 5B). Specifically, the contact angle values increased as the content of qDMAEMA increased (from 70° to 110°), resulting in a hydrophobic nature as a function of the long aliphatic chain that was introduced to the copolymers. Given these results, we managed to provide three P(HEMAx-co-DMAEMAy-qDMAEMAz) polymers with different hydrophobicity/hydrophilicity characteristics that will be studied furthermore in the form of cross-linked coatings in terms of their antibacterial activity.

**Scheme 3:**
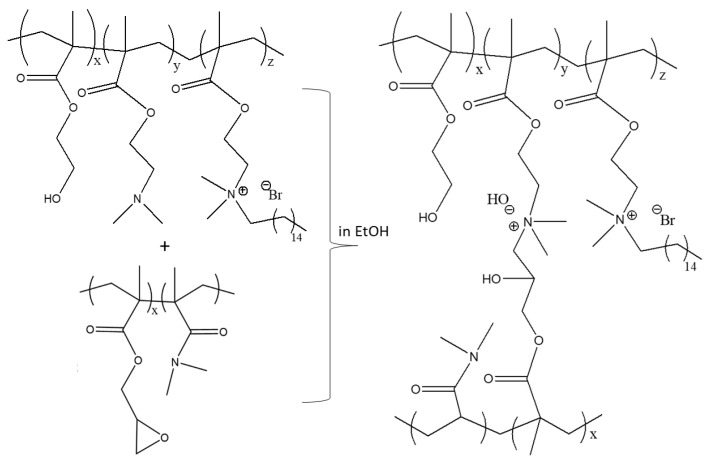
Cross-linking reaction between the P(HEMAx-co-DMAEMAy-co-qDMAEMAz)/P(DMAm-co-GMAx) copolymers.

The cationic terpolymers bearing quaternary hexadecylammonium groups were subsequently blended and cross-linked at 120 °C with P(DMAm-co-GMA42) copolymer to obtain stable coatings and membranes with possible antibacterial activity (Scheme 3). For this, P(HEMAx-co-DMAEMAy-co-qDMAEMAz) terpolymers with 70° and 92° contact angles (z = 13 and 40, respectively) were chosen to avoid high hydrophobicity levels in the resulting material. Thus, cross-linked polymeric membranes P(HEMAx-co-DMAEMAy-co-qDMAEMAz)/P(DMAm-co-GMAx) of a composition 70/30% *w*/*w* were prepared and studied in terms of stability and surface wettability.

**Figure 6:**
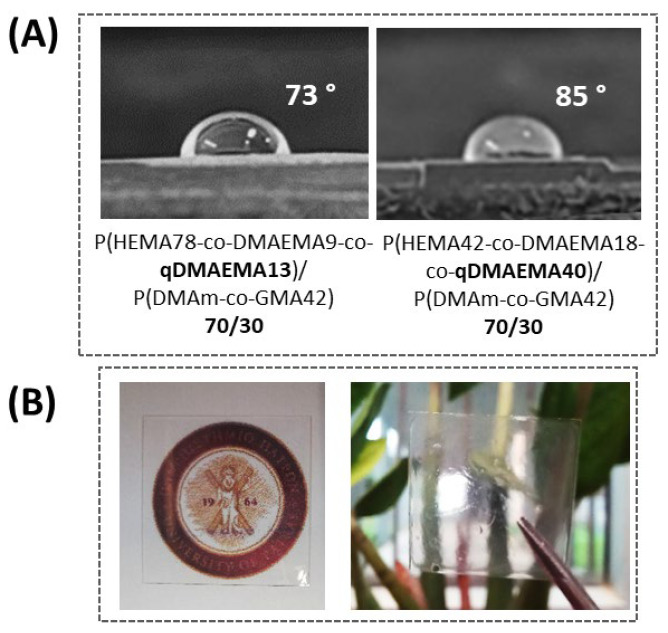
(**A**) Contact angle values of the cross-linked coatings P(HEMA78-co-DMAEMA9-co-qDMAEMA13)/P(DMAm-co-GMA42) and P(HEMA42-co-DMAEMA18-co-qDMAEMA40)/P(DMAm-co-GMA42) and (**B**) the appearance of the polymeric coatings on glass slides.

As shown in Table 3, the two cross-linked membranes remained highly stable when immersed in water for 1 day. For example, the membrane P(HEMA78-co-DMAEMA9-co-qDMAEMA13)/P(DMAm-co-GMA42) exhibited very low soluble fraction and water uptake values (3% and 47%, respectively) indicating the high cross-linking efficiency of this blend composition (eq_DMAEMA_/eq_GMA_: 1/2). Moreover, contact angle measurements (Figure 6A) revealed that after blending the quaternized terpolymers P(HEMAx-co-DMAEMAyco-qDMAEMAz) with the complementary copolymer P(DMAm-co-GMA42), hydrophobicity was maintained at low levels for both resulting coatings (namely, 73° and 85°). Thus, we managed to create stable polymeric materials that can form homogenous, transparent, and robust coatings with a balanced hydrophobicity/hydrophilicity, bearing cationic quaternary ammonium groups with long aliphatic chains (16 carbon atoms) for possible antimicrobial application (Figure 6B).

### 2.4. Assessment of Antibacterial Activity

**Figure 7:**
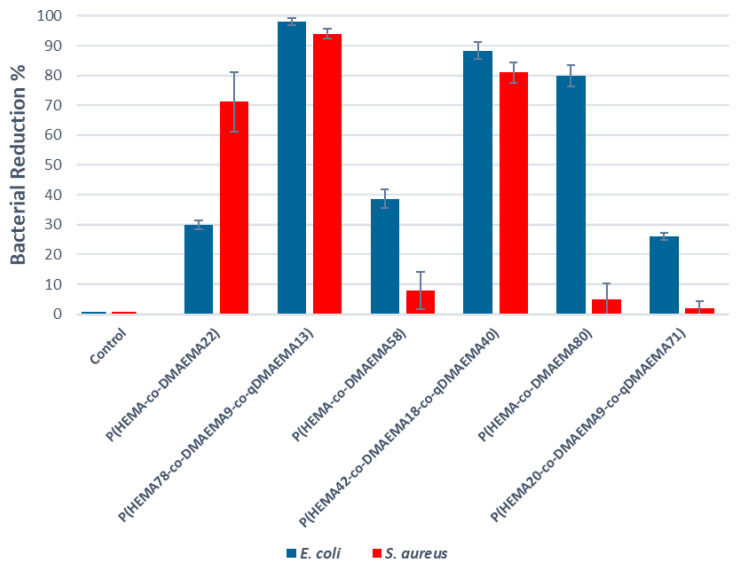
Cell reduction (%) for *E. coli* and *S. aureus* after contact (2 h) with the coatings of copolymers P(HEMA-co-DMAEMAx) and their quaternized analogs. Error bars represent standard deviation for three different measurements.

The positively charged nitrogen atoms of quaternary ammonium-based polymers may disrupt membranes, imitating antimicrobial peptides [33]. At first, these polymers may interact with the bacterial surfaces by electrostatic attractions of the charged species to enable their hydrophobic alkyl chains to penetrate the hydrophobic regions of the cell membranes through lipophilic interactions. The result is membrane disruption, which leads to bacterial death [33]. In the present work, the DMAEMA units were functionalized with hexadecyl chains for increased flexibility and hydrophobic effectiveness. Longer alkyl chains generally penetrate cell membranes better due to their hydrophobic properties [34]. However, excessive hydrophobicity can reduce the ability of the polymers to disrupt cell membranes due to increased aggregation between hydrophobic chains [35]. The creation of highly effective antimicrobial polymers based on quaternary ammonium compounds bearing hydrophobic moieties relies thus on achieving the right balance between overall hydrophobicity/hydrophilicity for effective bacterial death. Herein, this was accomplished by adjusting the hydrophilic and hydrophobic monomers’ composition as well as the degree of quaternization in the copolymers. The antimicrobial effectiveness of the synthesized polymeric materials was manifested as a reduction of cell populations after exposure to the polymers. The susceptibility of the bacterial strains *E. coli* and *S. aureus* is demonstrated in Figure 7 in relation to the composition of the synthesized copolymers (quaternized and non-quaternized).

The two bacterial species examined responded differently to the copolymers. In the case of non-quaternized copolymers P(HEMA-co-DMAEMAx), the increase in DMAEMA content (implying an increase in hydrophilicity) led to higher efficiency against *E. coli*. In contrast, the more hydrophobic copolymers favored the activity against *S. aureus*. The observed selectivity most likely stems from structural distinctions in the cell membranes of Gram-negative and Gram-positive bacteria: the cell wall of Gram-negative bacteria tends to be more anionic and hydrophilic in comparison to that of Gram-positive bacteria [36].

After the quaternization of the copolymers, different results were obtained. Overall, it was demonstrated that terpolymers modified with higher content of long alkyl chains (C16) exhibited notably lower effectiveness compared to those with fewer chains. Namely, the highest reduction level (98% and 94% for *E. coli* and *S. aureus*, respectively) was acquired in the case of quaternized P(HEMA78-co-DMAEMA9-co-qDMAEMA13) terpolymer, which had the lowest content of qDMAEMA.

**Figure 8:**
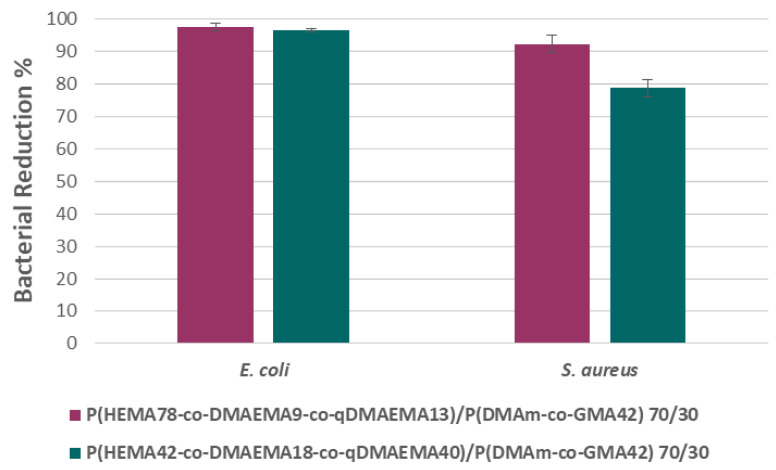
Cell reduction (%) for *E. coli* and *S. aureus* after contact (2 h) with the coatings of selected mixtures P(HEMAx-co-DMAEMAy-co-qDMAEMAz)/P(DMAm-co-GMA42) 70/30 *w*/*w*%. Error bars represent standard deviation for three different measurements.

Since the purpose of this work was the creation of stable antibacterial coatings, the two most efficient terpolymers were blended with the copolymer P(DMAm-co-GMA42) for cross-linking and were also tested for their killing efficacy. Even with a reduced proportion in the mixture (70%), the quaternized polymer retained its ability to eliminate both microorganisms (Figure 8). The cross-linked coatings with the smaller degree of quaternization (P(HEMA78-co-DMAEMA9-co-qDMAEMA13)) demonstrated great efficacy, achieving a 98.6% reduction in *E. coli* and 93.4% in *S. aureus*. Along with the surface wettability study above (Figure 6A), the less hydrophobic nature provided better results in killing efficacy. This observation is in qualitative agreement with previous studies discussing the antimicrobial properties of quaternized PDEAEMA brushes or blends [14,15]. Thus, the synergistic effect of the cationic nature of quaternized polymers and their hydrophilic/hydrophobic balance appears promising for developing innovative antimicrobial materials with superior robustness via cross-linking reaction.

### 2.5. Humidity-Response Performances

**Figure 9:**
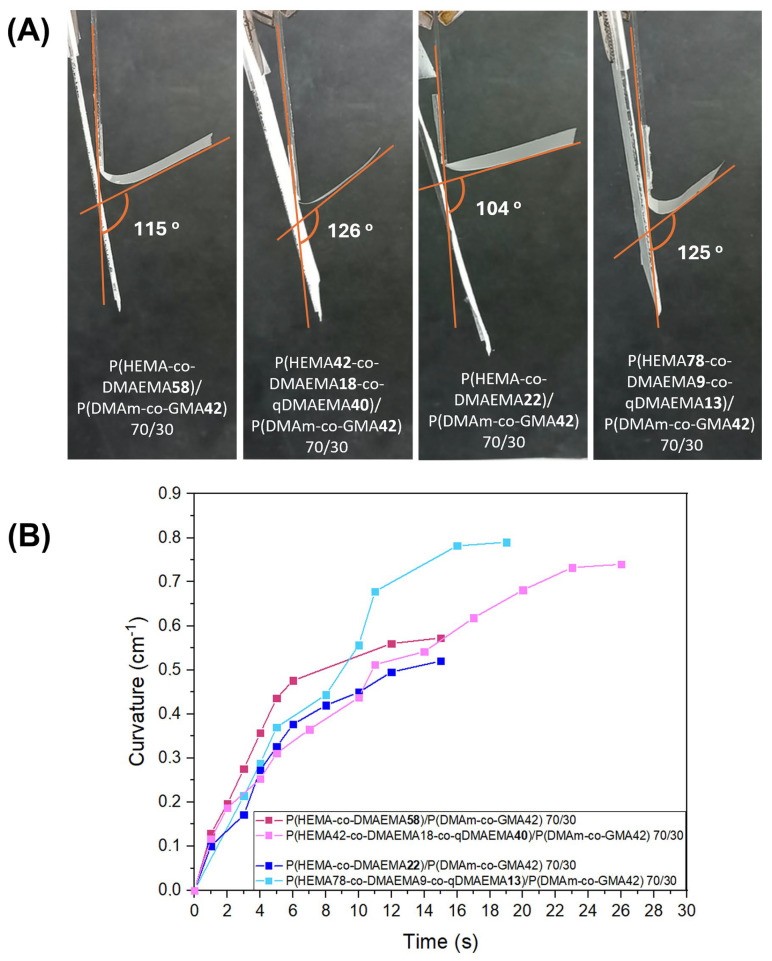
(**A**) Images of the cross-linked membranes P(HEMA-co-DMAEMAx)/P(DMAm-co-GMA42) 70/30 *w*/*w*% and their quaternized analogs, showing their bending angles when approaching a wet filter paper. (**B**) Calculated curvature of the above humidity-responsive membranes versus time.

During the characterization of the self-standing membranes obtained by the crosslinked polymer blends, the films underwent some spontaneous movements when placed on the palm of the hand. This behavior was further explored in relation to our previous findings, where humidity-driven mobility was found on completely different polymer blends [19]. To investigate how membranes respond to humidity, we initially measured the bending angle of narrow strip-shaped membranes when brought near a wet filter paper. The average membrane thickness was 40 μm. In Figure 9A, photographs (video snapshots of indicative Video S1 in Supplementary Materials) of each membrane in its maximum bending angle are shown. In general, all membranes exhibited high bending angles (104–125°) due to the hydrophilic groups they bear, so they interact with water molecules sufficiently. The bending movement of the membranes occurs due to the absorption of water molecules at the surface in contact with the wet filter paper (known as swelling behavior). As a possible explanation, one can consider that hydrogen bonds are formed between moisture and the hydrophilic groups of the copolymers (e.g., hydroxyl and amine groups), causing the expansion of polymeric chains and subsequent bending of the hydrated surface of the membrane. After the removal of the wet filter paper, the membrane strip returns to its original flat state, as shown in Video S1 (Supplementary Materials). This happens because the swollen hydrated membrane surface releases water molecules into the lower relative humidity environment.

Next, the curvature *K* (cm^−1^) of the membrane strips was calculated as reported previously [34]. Evolution of curvature values of the cross-linked membranes P(HEMA-co-DMAEMAx)/P(DMAm-co-GMA42) 70/30 *w*/*w*% and of their quaternized analogs are presented in the form of graphs in Figure 9B. In all cases, the actuation speed was approximately the same, whereas a slight distinction was observed regarding the maximum curvature values between the quaternized membranes and the non-quaternized ones. The membrane P(HEMA78-co-DMAEMA9co-qDMAEMA**13**)/P(DMAm-co-GMA42) exhibited the highest curvature in the shorter period at the same time (*K* = 0.8 s^−1^ at 16 s). Following, the quaternized membrane with a larger amount of hexadecyl-aliphatic chains, P(HEMA42-co-DMAEMA18-co-qDMAEMA**40**)/P(DMAm-co-GMA42), also exhibited high curvature values in 23 s. This behavior agrees with the influence of hydrophobicity on the humidity responsiveness of polymers.

**Figure 10:**
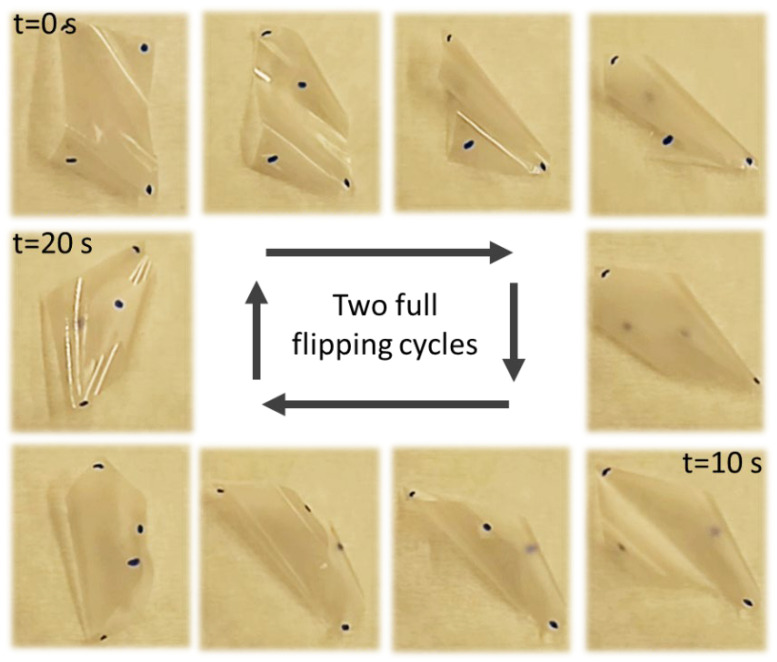
Photographs (video snapshots from Video S2 in Supplementary Materials) of the response of the P(HEMA-co-DMAΕΜA22)/P(DMAm-co-GMA42) 70/30 *w*/*w*% cross-linked membrane after contact with a wet filter paper.

The rectangular-shaped membranes P(HEMA78-co-DMAEMA9co-qDMAEMA**13**)/P(DMAm-co-GMA42) and P(HEMA-co-DMAEMA22)/P(DMAm-co-GMA42) showed an intriguing locomotive behavior consisting of several stages when placed on a damp surface, such as a pre-wetted filter paper (Figure 10 and Video S2 in Supplementary Materials). Each corner of the membranes used in this study was marked with a dot to aid in visualizing its motion. The observed mobility relies on a water vapor gradient, as discussed previously [18]. More precisely, upon contact, the lower side of the film, which is in contact with the moist substrate (higher humidity), rapidly experienced greater water-induced expansion compared to the side exposed to the air (lower humidity). Consequently, the film initially folded into a cylindrical structure, and as the center of gravity shifted, the film toppled over, causing its upper surface to flip downwards. Subsequently, the film bent from one corner, rolled horizontally, and initiated a new flipping cycle. The asymmetric swelling may result from the spontaneous formation of a bilayer structure that propels the continuous flipping motion of the film. Upon removal from the moist substrate, the film’s motion ceases instantly and can be restarted by placing it back on the moist paper. As can be seen in Figure 10, the cross-linked membrane P(HEMA-co-DMAΕΜA22)/P(DMAm-co-GMA42) 70/30 *w*/*w*% performed a full cycle of flipping motion in approximately 10 s. It is noteworthy that the respective quaternized membrane, P(HEMA78-co-DMAEMA9co-qDMAEMA**13**)/P(DMAm-co-GMA42), presented this humidity-driven behavior much faster (Video S2 of Supplementary Materials). This observation suggests that the hydrophobic parts may promote the desorption of water molecules in the recovery process, leading to accelerated recovery and enhanced reversibility.

**Figure 11:**
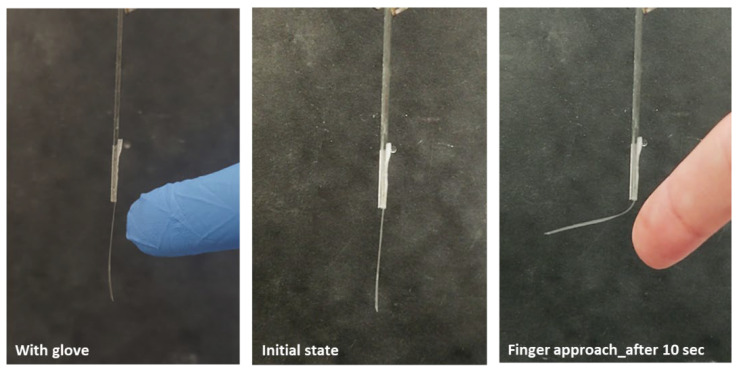
Photographs (snapshots from Video S5 in Supplementary Materials) of the response of the P(HEMA-co-DMAΕΜA22)/P(DMAm-co-GMA42) 70/30 *w*/*w*% cross-linked membrane, when a finger approaches.

Moisture from the palm of a hand could also provoke this kind of locomotion to the membranes (Videos S3 and S4 in Supplementary Materials). The actuation speed was different between the two membranes in this experiment as well. The flipping performance was faster and had higher repeatability on the palm than on the wet paper. This is probably due to the difference in the humidity gradient. Since the hand is warmer (~37 °C) than the wet filter paper (~22 °C), it is likely to contain more water vapors, leading to a higher content of humidity and, thus, a higher humidity gradient. It is remarkable that humidity responsiveness can be performed contactless. This is demonstrated in Figure 11, where a rectangular strip of the P(HEMA-co-DMAΕΜA22)/P(DMAm-co-GMA42) 70/30 *w*/*w*% cross-linked membrane exhibited a rapid response when exposed to finger humidity. The proximity of a naked finger to one side of the film caused bending of about 90° without external force. Humidity is necessary since no movement was observed when the finger was covered by a glove. 

## 3. Materials and Methods

### 3.1. Materials

Glycidyl methacrylate (GMA), *N*,*N*-dimethylacrylamide (DMAm) 2-(dimethylamino)ethyl methacrylate (DMAEMA), 2-hydroxyethylmethacrylate (HEMA), the initiator azobisisobutyronitrile (AIBN), 1-bromohexadecane, as well as deuterated methanol (MeOD) and deuterated chloroform (CDCl_3_) were procured from Sigma-Aldrich Chemie GmbH, Taufkirchen, Germany and utilized without further purification. *N*,*N*-dimethylformamide (DMF), tetrahydrofuran (THF), diethyl ether, and hexane were obtained from Fischer and employed as received. Ethanol was sourced from Honeywell. Ultrapure water was generated using an SG apparatus water purification unit.

### 3.2. Synthesis of P(HEMA-co-DMAEMAx) Copolymers

The copolymers poly(2-hydroxyethylmethacrylate-co-2-(dimethylamino)ethyl methacrylate) were synthesized through free radical polymerization using AIBN (1 mol% of the total monomers’ mole) as initiator and DMF (30% *w*/*v*) as solvent. The desired amounts of the two monomers (total monomer concentration 1 M) were dissolved in the DMF, the solution was degassed, and the initiator was added. The reaction was left to proceed under vigorous stirring in a nitrogen atmosphere in an oil bath set at 70 °C for 48 h. The final products were purified and received through dialysis against ultra-pure water (using a dialysis membrane with an MWCO of 12,000–14,000 Da) and freeze drying, respectively. Copolymers with 22 to 80 mol% DMAEMA were synthesized. The actual mol fractions of HEMA and DMAEMA were calculated from proton nuclear magnetic resonance (^1^H-NMR), using MeOD as solvent. For comparison reasons, homopolymers were also synthesized according to the literature [31,37]. The copolymers are denoted as P(HEMA-co-DMAEMAx), where x is the mol fraction of DMAEMA units as determined from ^1^H-NMR, as shown in Table 4.

### 3.3. Synthesis of P(DMAm-co-GMAx) Copolymers

The copolymers poly(*N*,*N*-dimethylacrylamide-co-glycidyl methacrylate) were synthesized as reported elsewhere [32], via free radical polymerization in THF with AΙΒΝ as initiator. The actual mol fractions of the moieties in the copolymers were determined through ^1^H-NMR, using CDCl_3_ as solvent. The copolymers are represented as P(DMAm-co-GMAx), where x is the mol fraction of GMA units (x = 23 or 42%) as determined from ^1^H-NMR, as shown in Table 5.

### 3.4. Post-Polymerization Quaternization

The P(HEMA-co-DMAEMAx) copolymers underwent a reaction with an alkyl halide, namely 1-bromodecahexane, at 60 °C, resulting in the formation of P(HEMAx-co-DMAEMAy-co-qDMAEMAz) copolymers. The reaction took place in a round-bottom flask, using DMF as the solvent and P(HEMA-co-DMAEMAx) copolymers at 10 wt% concentration. The degree of quaternization was adjusted between 5 and 15% by varying the molar ratio of the alkyl halide to the DMAEMA repeating units. Characterization of the quaternized copolymers was performed using ^1^H-NMR spectroscopy to ascertain the actual degree of quaternization.

### 3.5. Preparation of Cross-Linked Membranes and Coatings

Following the cross-linking reaction between amine and epoxy groups, membranes and coatings of the complementary copolymers were fabricated using the solution casting method. In this process, P(HEMA-co-DMAEMAx) and P(DMAm-co-GMAx) copolymers were initially dissolved in a mixture of solvents water/ethanol 1/1 at a concentration of 5 *w*/*v*%. The solutions were then mixed at the desired ratio, and the resulting blends were stirred until complete homogenization was achieved. Subsequently, the blends were cast onto polystyrene dishes (for membrane preparation) or onto small glass coupons (for coating preparation) and were allowed to undergo solvent evaporation at room temperature for 24 h. Following this, the formed membranes and coatings were placed in an oven at two different temperatures, 80 °C and 120 °C, for 24 h for cross-linking reaction to occur. Various blend compositions (*w*/*w*%) of the two copolymers were experimented with to determine the optimal cross-linking conditions, and the tested parameters are detailed in Table 3. The same procedures were followed for the preparation of the P(HEMAx-co-DMAEMAy-co-qDMAEMAz)/P(DMAm-co-GMAx) membranes and coatings as well.

### 3.6. Chemical Characterization

#### 3.6.1. Proton Nuclear Magnetic Resonance (^1^H-NMR)

^1^H-NMR spectra of P(HEMA-co-DMAEMAx), their quaternized analogs, and P(DMAm-co-GMAx) copolymers in MeOD and CDCl_3_, respectively, were obtained at 400 MHz and at 25 °C using a Bruker AVANCE DPX 400 spectrometer (Bruker BioSpin GmbH, Magnet Division, Karlsruhe, Germany).

#### 3.6.2. Attenuated Total Reflection Fourier Transform Infrared Spectroscopy (ATR-FTIR)

The ATR-FTIR spectra of the copolymers, homopolymers, and cross-linked membranes were recorded on a Bruker Platinum ATR-FTIR spectrometer (Bruker Optics GmbH, Ettlingen, Germany).

### 3.7. Physicochemical Characterization

#### 3.7.1. Soluble Fraction and Water Uptake Studies

Small sections of the prepared membranes cast in different temperatures (RT, 80 °C, and 120 °C) were pre-weighed and then immersed in glass vials with water and left at room temperature overnight. The soluble fraction of the membranes was evaluated gravimetrically using the following equation:

(1) $$\mathrm{Soluble}\;\mathrm{Fraction}\;(\%\;\mathrm{wt}.)=\frac{\left|W-W_{0}\right|}{W_{0}}\;\%$$

where W_0_ and W are the measured weights of the dried membranes before and after immersion in the solvents, respectively. 

For the calculation of the water uptake, right after their removal from the vials, the membranes were lightly wiped with filter paper and weighed in their wet form. The same equation mentioned above (1) was used to calculate the solvent uptake, where W is the measured weight of the wet membranes after their immersion in water.

#### 3.7.2. Contact Angle Measurements

Contact-angle measurements were conducted using the sessile drop method. In detail, a 10 μL droplet of ultrapure water was dispensed onto the surfaces of the polymeric coatings, and the contact angles were determined using ImageJ software 1.53t.

### 3.8. Antimicrobial Activity Assay

#### 3.8.1. Bacterial Culture Preparation

Gram-negative *Escherichia coli* (*E. coli*) 9001 and Gram-positive *Staphylococcus aureus* (*S. aureus*) NCTC 6571 were used to assess the antimicrobial efficacy of the polymers synthesized in this work and their corresponding cross-linked coatings. The aforementioned strains were obtained from the Health Protection Agency, Porton Down, Salisbury, U.K. Individual colonies of each strain were cultivated overnight (18 h) in 8 mL of LB broth in 15 mL tubes positioned horizontally at 80 rpm and 37 °C, resulting in a final cell density of approximately 10^8^–10^9^ cfu × mL^−1^.

#### 3.8.2. Bacterial Reduction Assay

Under aseptic conditions, the polymer solutions were applied on glass slides (18 mm × 18 mm) and allowed to dry at 120 °C to obtain stable coatings. Subsequently, 20 μL aliquots from overnight bacterial cultures were deposited on the coated glass slides and incubated at 22 °C for 120 min. The slides were then immersed four times in LB medium (30 mL of LB in 50 mL tubes) to wash off the bacteria, and the cultures were horizontally positioned and incubated at 80 rpm, 37 °C for varying durations depending on the bacterial strains (5 h for *E. coli* and 16 h for *S. aureus*). Optical density measurements at 600 nm (A_600_) were obtained to assess the cell growth (scattering), with sample dilution in water (if necessary), to ensure a final A_600_ value of ≤0.5. The inoculation volume (20 μL) and growth duration for each species were selected to align with the exponential growth phase of control cultures (without polymer exposure). All experiments were conducted in triplicate on different days, each with distinct starting bacterial cultures/strains. The impact of polymers on cell reduction was quantified using the following equation:

(2) $$Cell\;Reduction\;\%=\;\frac{{A_{600}\;Control-A}_{600}\;Sample}{A_{600}\;Control}\;\times 100\;\%$$

where A_600_ Control are the values of the control cultures (without polymer exposure), whereas A_600_ Sample are the values of those exposed to the polymers. In the graphs presented in the Section 2, the values represent the averages of three experiments, and the error bars indicate the standard deviation for these means.

### 3.9. Humidity-Driven Curvature Measurements

Two glass slides placed parallel to each other were used for this experiment. One slide held the membrane strip fixed at the lower end, while the opposite slide was covered with a damp filter paper aligned parallel to the hanging membrane strip. The average membrane thickness was 40 μm. The moisture level on the filter paper was determined to be around 62% using the oven-dry method. This setup allowed the membrane strip to move freely. As the wet filter paper approached the strip, it caused the strip to bend in the opposite direction. After reaching a stable maximum bending position, photographs were captured to measure the bending angle using ImageJ software. Subsequently, the curvature (represented as K) was calculated using the equation reported in the literature [38]:

(3) K=a×π/180°×L

where a (°) denotes the bending angle and L (cm) refers to the length of the free section of the curved membrane. An indicative video of the experiment (Video S1) is presented in Supplementary Materials.

## 4. Conclusions

This work demonstrates a straightforward and environmentally more acceptable methodology for creating stable antibacterial coatings and membranes by employing a cross-linking reaction involving amphiphilic copolymers containing quaternary hexadecylammonium groups at various compositions, along with complementary reactive groups. The primary objective was the synthesis of a series of complementary reactive copolymers P(HEMA-co-DMAEMAx) and P(DMAm-co-GMAx) and the partial quaternization with hexadecyl groups of DMAEMA units at varying levels to assess the impact of the cross-linking conditions and the ratio of reactive groups of the two copolymers (mol DMAEMA/mol GMA) on the antimicrobial efficacy and stability of the resulting membranes. Three P(HEMAx-co-DMAEMAy-qDMAEMAz) terpolymers were synthesized with different hydrophobicity/hydrophilicity characteristics and were further studied in the form of coatings cross-linked with P(DMAm-co-GMA42). The derived coatings exhibited high antibacterial activity against *E. coli* and *S. aureus*. The results of the present study provide useful insight into the impact of the balance between hydrophobicity and hydrophilicity on the effectiveness of cationic antibacterial polymeric materials. Moreover, the cross-linked membranes developed in this study were highly responsive to wet surfaces. When placed on a wet filter paper or on the palm of a hand, they exhibited immediate responses through folding and flipping motions of great repeatability. These stable, self-supporting, and humidity-responsive monolayer membranes, coupled with their significant antimicrobial effectiveness, could be promising candidates for practical utilization in various fields, including the development of biomedical sensors and smart devices for regulated therapy and diagnostics. Notably, the humidity-responsive properties do not necessarily involve contact, widening thus the spectrum of potential “smart” applications, including, for instance, the construction of non-contact antimicrobial switches.

## Figures and Tables

**Table 1:** The membranes of various polymeric blends of P(HEMA-co-DMAEMAx)/P(DMAm-co-GMAx) at different % (*w*/*w*) and eq_DMAEMA_/eq_GMA_ compositions.

Complementary Copolymers	Composition % (*w*/*w*)	eq_DMAEMA_/eq_GMA_	Membrane
P(HEMA-co-DMAEMA**80**)/ P(DMAm-co-GMA42)	50/50	1.5/1	M1
	70/30	3.5/1	M2
P(HEMA-co-DMAEMA**58**)/ P(DMAm-co-GMA42)	50/50	1.2/1	M3
	70/30	2.8/1	M4
P(HEMA-co-DMAEMA**40**)/ P(DMAm-co-GMA42)	50/50	0.8/1	M5
	70/30	2/1	M6
P(HEMA-co-DMAEMA**22**)/ P(DMAm-co-GMA40)	50/50	0.4/1	M7
	70/30	1/1	M8

P(HEMA-co-DMAEMA**80**)/ P(DMAm-co-GMA23)	50/50	2.8/1	M9
	70/30	6.6/1	M10
P(HEMA-co-DMAEMA**58**)/ P(DMAm-co-GMA23)	50/50	2.2/1	M11
	70/30	5/1	M12
P(HEMA-co-DMAEMA**40**)/ P(DMAm-co-GMA23)	50/50	1.5/1	M13
	70/30	3.6/1	M14
P(HEMA-co-DMAEMA**22**)/ P(DMAm-co-GMA23)	50/50	0.8/1	M15
	70/30	2/1	M16

**Table 2:** Quaternization degree of the P(HEMA-co-DMAEMAx) copolymers of different compositions.

Copolymers	Feed Composition (%mol qDMAEMA)	^1^H-NMR Composition (%mol qDMAEMA)	Quaternized Copolymers
P(HEMA-co-DMAEMA22)	16	13	P(HEMA78-co-DMAEMA9-co-qDMAEMA13)
P(HEMA-co-DMAEMA58)	48	40	P(HEMA42-co-DMAEMA18-co-qDMAEMA40)
P(HEMA-co-DMAEMA80)	80	71	P(HEMA20-co-DMAEMA9-co-qDMAEMA71)

**Table 3:** The membranes of selected polymeric blends of P(HEMAx-co-DMAEMAy-co-DMAEMAz)/P(DMAm-co-GMA42) of 70/30% (*w*/*w*) composition and their soluble fraction and water uptake measurements.

Complementary Copolymers	Composition % (*w*/*w*)	eq_DMAEMA_/eq_GMA_	T (°C)	Soluble Fraction %	Water Uptake %
P(HEMA78-co-DMAEMA9co-qDMAEMA**13**)/ P(DMAm-co-GMA42)	70/30	1/2	120	3	47
P(HEMA42-co-DMAEMA18-co-qDMAEMA**40**)/ P(DMAm-co-GMA42)	70/30	1/3	120	10	209

**Table 4:** P(HEMA-co-DMAEMAx) copolymers synthesized in this work with different monomers’ compositions.

Copolymers	Feed Composition (%mol DMAEMA)	^1^H-NMR Composition (%mol DMAEMA)
P(HEMA-co-DMAEMA22)	20	22
P(HEMA-co-DMAEMA40)	40	40
P(HEMA-co-DMAEMA58)	60	58
P(HEMA-co-DMAEMA80)	80	80

**Table 5:** P(DMAm-co-GMAx) copolymers synthesized in this work with different monomers’ compositions.

Copolymers	Feed Composition (%mol GMA)	^1^H-NMR Composition (%mol GMA)
P(DMAm-co-GMA23)	20	23
P(DMAm-co-GMA42)	40	42

## Data Availability

The data presented in this study are available in article and Supplementary Materials.

## Supplementary Materials

The following supporting information can be downloaded at https://www.mdpi.com/article/10.3390/molecules29102372/s1: characterization of the P(DMAm-co-GMAx) copolymers through ^1^H-NMR and ATR-FTIR spectroscopies, soluble fraction, and water uptake results of the polymeric membranes (PDF); Figure S1. (A) 1H-NMR spectra in CDCl3 and (B) ATR-FTIR spectra of the P(DMAm-co-GMAx) copolymers synthesized in this work, as well as of the respective homopolymers PDMAm and PGMA, for comparison, Figure S2. (A) Soluble fraction and (B) water uptake % of the crosslinked membranes P(HEMA-co-DMAEMAx)/P(DMAm-co-GMA23) in different temperatures. (C) Soluble fraction and (D) water uptake % of the crosslinked membranes P(HEMA-co-DMAEMAx)/P(DMAm-co-GMA42) in different temperatures; movie file showing the humidity-driven bending mobility of the membrane P(HEMA-co-DMAΕΜA58)/P(DMAm-co-GMA42) 70/30 when approaching a wet filter-paper (Video S1); movie file showing the flipping mobility of the cross-linked membranes P(HEMA-co-DMAΕΜA22)/P(DMAm-co-GMA42) 70/30 and P(HEMA-co-DMAΕΜA9-co-qDMAEMA13)/P(DMAm-co-GMA42) 70/30, upon contact with a wet filter paper (Video S2); movie file showing the humidity-driven mobility of the membrane P(HEMA-co-DMAΕΜA9-co-qDMAEMA13)/P(DMAm-co-GMA42) 70/30 when placed on the palm of a hand (Video S3); movie file showing the humidity-driven mobility of the membrane P(HEMA-co-DMAΕΜA22)/P(DMAm-co-GMA42) 70/30 when placed on the palm of a hand (Video S4); movie file showing the humidity-driven mobility of the membrane P(HEMA-co-DMAΕΜA22)/P(DMAm-co-GMA42) 70/30 when a naked finger approaches (Video S5).
