# Supplementary material for: Facile Synthesis of Dual-Functional Cross-Linked Membranes with Contact-Killing Antimicrobial Properties and Humidity-Response
Source: Molecules. 2024 May 17;29(10):2372. doi: 10.3390/molecules29102372 (PMC11123689; doi:10.3390/molecules29102372)
Supplement: Supplementary file 1 [file molecules-29-02372-s001.zip › Supporting Information.pdf]

Supporting Information

Facile synthesis of dual-functional cross-linked membranes with contact-killing antimicrobial properties and humidity-response

Ioanna Tzoumani [1]*, Denisa Druvari [1], Miltiadis Evangelidis [1], Alexios Vlamis-Gardikas [1], Georgios Bokias [1] and Joannis K. Kallitsis [1,2,]*

[1] Department of Chemistry, University of Patras, GR–26504, Patras, Greece; tzoumani@ac.upatras.gr (I.T.); druvari@upatras.gr (D.D.); up1073714@ac.upatras.gr; avlamis@upatras.gr (A.V.-G.); bokias@upatras.gr (G.B.)

[2] Foundation for Research and Technology-Hellas, Institute of Chemical Engineering Sciences (FORTH/ICE-HT), Stadiou Str, GR–26504 Rio-Patras, Greece

* Correspondence: kallitsi@upatras.gr (J.K.K.), +30-26-1096-2952 (J.K.K.), tzoumani@ac.upatras.gr (I.T.) +30-26-1099-7121 (I.T.)

1.1 Characterization of the P(DMAm-co-GMAx) copolymers

Figure S1A shows the ^{1}H-NMR spectra of the P(DMAm-co-GMAx) copolymers in $CDCl_3$. For comparison, the corresponding spectra of the homopolymers PDMAm and PGMA are also shown. The presence of methyl protons ($-CH_3$, d) of GMA is shown at 0.92 ppm. The methyl protons of the polymeric backbone, ($-CH_2-$, a, c) and ($-CH_2$, b), of both DMAm and GMA units appear at 1.10-2.10 ppm and 2.65 ppm respectively. Additionally, the peaks at 3.80 and 4.35 ppm correspond to the methylene protons ($-CH_2-O$, f) of GMA. The peak observed at 3.10 ppm is due to the methine proton (g) of GMA, while the large peak at 2.90 ppm corresponds to the methyl groups (e) of DMAm and the methylene protons (h) of the epoxy ring of GMA as well.

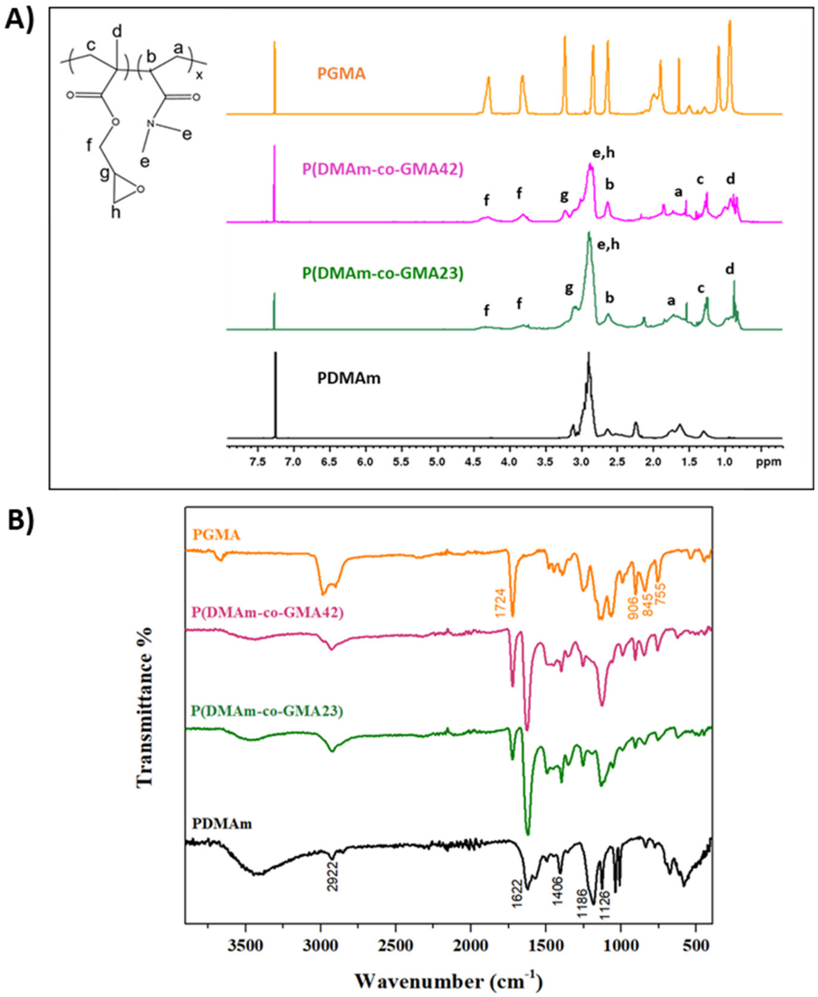

Figure S1. A) ^{1}H-NMR spectra in $CDCl_3$ and B) ATR-FTIR spectra of the P(DMAm-co-GMAx) copolymers synthesized in this work, as well as of the respective homopolymers PDMAm and PGMA, for comparison.

The synthesized copolymers were further examined by ATR-FTIR spectroscopy, and the spectra are shown in Figure S1B. For comparison, the spectra of PDMAm and PGMA homopolymers are also presented. In particular, the peak at 1622 cm^{-1} is attributed to the C=O vibrations of the amide, while the smaller one at 1406 cm^{-1} is attributed to the C-N vibrations. The peak at 2922 cm^{-1} is attributed to the C-H bond vibrations of the methyl groups of the copolymer's backbone. The peaks located at 1186 and 1126 cm^{-1} are attributed to the N-H and C-N bonds stretching vibrations. The existence of the GMA unit in P(DMAm-co-GMAx) copolymers is confirmed by the characteristic peaks at 845 and 906 cm^{-1} attributed to the epoxy ring vibrations and the peak at 1252 cm^{-1} attributed to the C-H bond of the epoxy group as well. The peak at 755 cm^{-1} corresponds to the bending vibration of the C-H group of the epoxy ring. In addition, in the region of 1724 cm^{-1} the characteristic stretching vibration the carbonyl group (C=O) of GMA is observed.

1.2 Soluble fraction and water uptake results

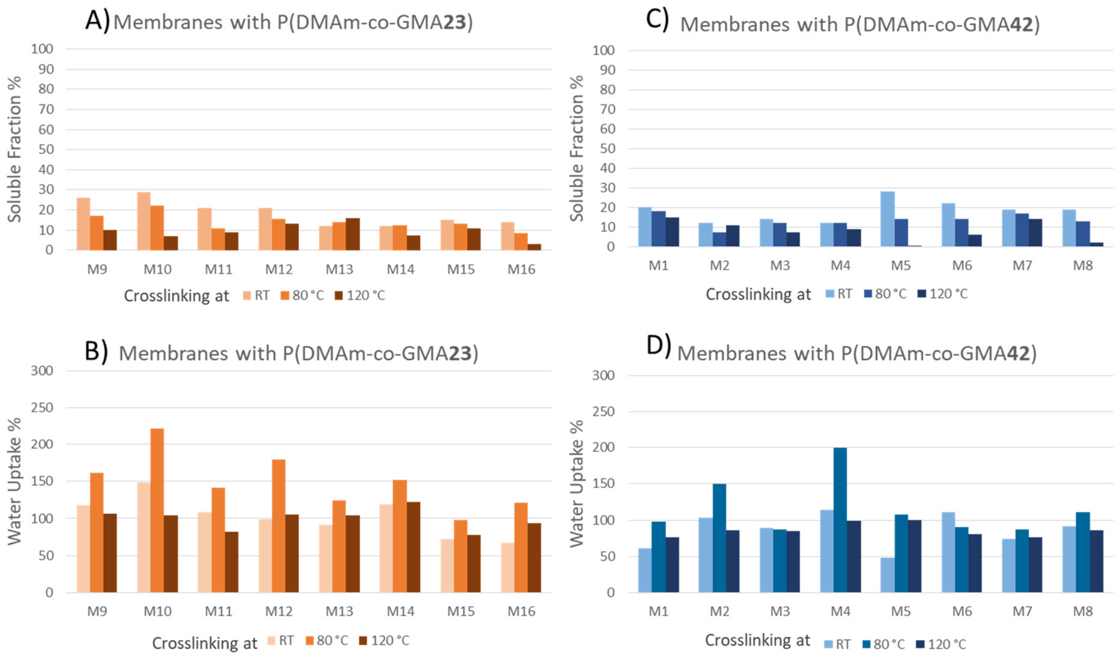

Figure S2. (A) Soluble fraction and (B) water uptake % of the crosslinked membranes P(HEMA-co-DMAEMAx)/P(DMAm-co-GMA**23**) in different temperatures. (C) Soluble fraction and (D) water uptake % of the crosslinked membranes P(HEMA-co-DMAEMAx)/P(DMAm-co-GMA**42**) in different temperatures.
