# Supplementary figures and images for: Facile Synthesis of Dual-Functional Cross-Linked Membranes with Contact-Killing Antimicrobial Properties and Humidity-Response
Source: Molecules. 2024 May 17;29(10):2372. doi: 10.3390/molecules29102372 (PMC11123689; doi:10.3390/molecules29102372)

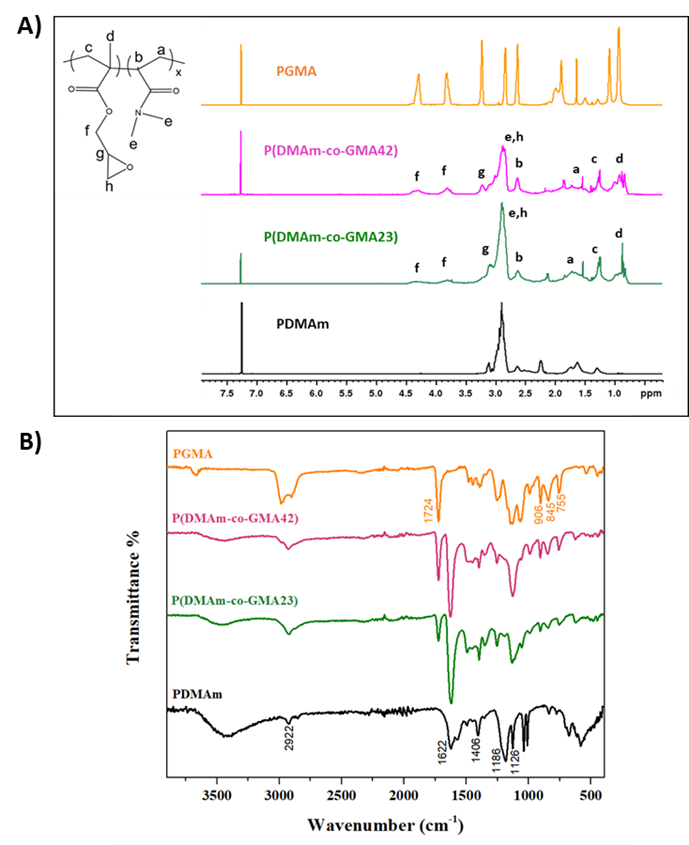

Supplement: Supplementary file 1 [file molecules-29-02372-s001.zip › Figure S1.tif]

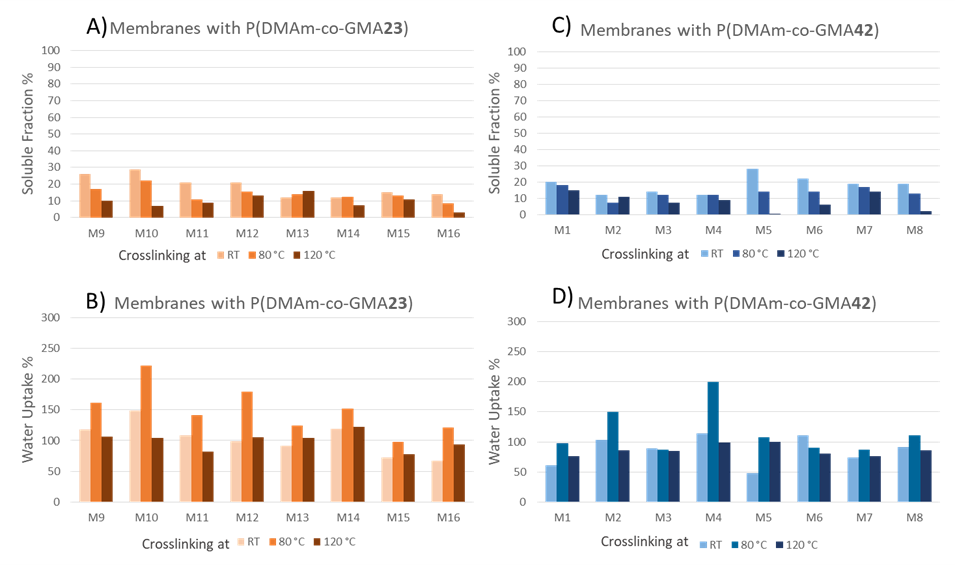

Supplement: Supplementary file 1 [file molecules-29-02372-s001.zip › Figure S2.tif]
